# The EPSRC’s Policy of Responsible Innovation from a Trading Zones Perspective

**DOI:** 10.1007/s11024-016-9294-9

**Published:** 2016-03-23

**Authors:** Joseph Murphy, Sarah Parry, John Walls

**Affiliations:** School of Interdisciplinary Studies, University of Glasgow, Dumfries Campus, Rutherford/McCowan Building, Bankend Road, Dumfries, DG1 4ZL Scotland, UK; Science, Technology and Innovation Studies (STIS), University of Edinburgh, High School Yards, Edinburgh, EH1 1LZ Scotland, UK

**Keywords:** Responsible innovation, Trading zone, Language, Expertise, EPSRC, Geoengineering

## Abstract

Responsible innovation (RI) is gathering momentum as an academic and policy debate linking science and society. Advocates of RI in research policy argue that scientific research should be opened up at an early stage so that many actors and issues can steer innovation trajectories. If this is done, they suggest, new technologies will be more responsible in different ways, better aligned with what society wants, and mistakes of the past will be avoided. This paper analyses the dynamics of RI in policy and practice and makes recommendations for future development. More specifically, we draw on the theory of ‘trading zones’ developed by Peter Galison and use it to analyse two related processes: (i) the development and inclusion of RI in research policy at the UK’s Engineering and Physical Sciences Research Council (EPSRC); (ii) the implementation of RI in relation to the Stratospheric Particle Injection for Climate Engineering (SPICE) project. Our analysis reveals an RI trading zone comprised of three quasi-autonomous traditions of the research domain – applied science, social science and research policy. It also shows how language and expertise are linking and coordinating these traditions in ways shaped by local conditions and the wider context of research. Building on such insights, we argue that a sensible goal for RI policy and practice at this stage is better local coordination of those involved and we suggest ways how this might be achieved.

## Introduction

Responsible innovation (RI) has been defined as “… taking care of the future through collective stewardship of science and innovation in the present” (Stilgoe et al. 2013: 3). Building on debates in science, technology and innovation studies, commercial and regulatory experience, advocates argue that a range of tools and techniques should be used to open up scientific research at an early stage so that many actors and issues can steer innovation trajectories. If this is done, they suggest, new technologies will be more responsible in different ways, better aligned with what society wants, and mistakes of the past will be avoided. In practice this means exposing scientific research to a wider range of influences, beyond, for example, risk assessment, ethical approval or commercial appraisal. Ultimately, Guston (2007: 300) argues, RI involves shaping science and innovation with the aim of “… helping people pursue more uplifting lives in more just societies”.

The RI debate emerged in the 2000s and is now a key academic debate and policy agenda linking science and society. Hellström (2003) made an early contribution exploring RI as a way to assess and manage systemic innovations and ‘negative synergies’. Soon after, Guston (2004, 2007) made a valuable institutional argument by emphasising the need for responsible innovation centres in increasingly commercialised universities. More recently the focus shifted to implementation in projects (e.g. Owen and Goldberg 2010). Technologies in relation to which RI has been developed include nanotechnology, synthetic biology and ICTs (information and communication technologies), and recent significant developments include an edited book (Owen et al. 2013), launch of the *Journal of Responsible Innovation* (2014), and embedding in policy (Sutcliffe 2011; von Schomberg 2011; European Commission 2013). As this summary suggests, there are different variants of RI, rather than one canonical account, and these diverge and overlap in significant ways. In this paper we focus on one variant whilst occasionally referring to others and highlighting similarities and differences where this is useful.

The aim of this paper is to understand the dynamics of RI in one setting in order to make recommendations regarding further development. To this end we address two key questions: (Q1) How is RI linking the applied sciences, social sciences and research funders? (Q2) How should the policy and practice of RI be taken forward? These questions are important because the existing literature tends to focus on understanding tools and techniques of RI – such as mid-stream modulation and risk registers – rather than trying to understand in a broader sense how those involved are interacting. And yet our research highlights complex and important issues around the dynamics of interaction which have implications for policy and practice.

To answer our key questions we draw on the theory of ‘trading zones’ (TZs) developed by Peter Galison (1996, 1997, 1999, 2010) to explain interactions between different traditions or subcultures of physics across the 20^th^ century. As Kellog et al. (2006: 39) have argued: “Importantly, this metaphor is not intended to evoke the commodified transactions of efficient markets, but the complex interactions of distinct communities encountering each other for purposes of exchange”. We also draw on the work of scholars who have extended TZs research theoretically (in relation to expertise and authority), empirically (beyond physics to non-science disciplines and policymaking) and normatively (to facilitate or encourage particular kinds of interactions).

RI is a good case to explore from a TZs perspective because it involves interactions between distinct traditions or sub-cultures of the research domain. This helps to explain why others have already linked RI and the TZs approach. For example, Nerlich (2014) has argued that:

*Once widely spread, buzzwords [like responsible innovation] establish something like a ‘trading zone’ in which people from different backgrounds… can communicate without however having to be too explicit about what they are saying.*

With a somewhat more normative agenda, Gorman et al. (2009: 185) examine the role of ethicists in nanotechnology research and argue for “The establishment of a trading zone coupled with moral imagination” to facilitate collaboration and effective communication (see also Gorman et al. 2013). We build on these and other contributions below.

Our analysis focuses on two related processes central to the evolution of one variant of RI: (i) the development and inclusion of RI in research policy at the UK’s Engineering and Physical Sciences Research Council (EPSRC); (ii) the implementation of RI in relation to the Stratospheric Particle Injection for Climate Engineering (SPICE) project. There have been significant developments elsewhere (particularly in the United States and European Union) but we focus on the EPSRC and SPICE for a number reasons. For example, the EPSRC is the most important funder of applied scientific research in the UK and a key site where advocates of RI have made progress. Other organisations are also learning about RI from the EPSRC. And SPICE is important because it has emerged as an important example of RI in practice (Macnaghten and Owen 2011; Stilgoe et al. 2013; Stilgoe 2015). However, the only critical reflections available on the EPSRC-RI-SPICE case are provided by scholars who were directly involved and this makes analysis by others a priority.

The paper draws on research and engagement in three areas: First, a comprehensive review of academic and policy literature relating to TZs, RI (focusing on the EPSRC) and the SPICE project; Second, interviews with key actors involved in developing, promoting and implementing RI in the EPSRC and in relation to SPICE; Third, a one-day workshop on RI we organised which brought together applied scientists, social scientists and representatives of UK research councils, including the EPSRC, BBSRC (Biotechnology and Biological Sciences Research Council) and TSB (Technology Strategy Board).

The following section summarises the theory of TZs. Subsequently, we describe the development and implementation of RI in the EPSRC and in relation to the SPICE project. Q1 will then be answered by using the theory of TZs to analyse RI in policy and practice. In the conclusion we answer Q2 by moving from analytical to normative application of TZs theory.

## Trading Zones

Peter Galison (1997: xvii) describes *Image and Logic: A Material Culture of Microphysics* as “… a back-and-forth walk through physics to explore the site where engine grease meets up with experimental results and theoretical constructions”. Put another way, it is a journey to discover how distinct branches of physics interacted across the 20^th^ century. His answer is the theory of ‘trading zones’ (TZs) and towards the end of *Image and Logic* he defines a TZ as “… the site – partly symbolic and partly spatial – at which the local coordination between beliefs and actions takes place” (Galison 1997: 784). In this section we summarise the theory and explore how others have extended it.

### Traditions and Languages

Galison’s work builds on an analysis of interactions around detectors like radar and bubble chambers – “… those objects large and small that mediate between the microworld and the world of knowledge” (Galison 1997: xviii). From this he distils three key insights and constructs a heuristic device. First, 20^th^ century physics was comprised of at least three ‘subcultures’ or ‘quasi-autonomous traditions’ – theory, experimentation and instrument building. Second, each tradition evolved through distinct periods over time (flux-stability-flux) but breaks between periods in the different traditions did not necessarily coincide. Third, the traditions were intercalated, meaning that they “… coordinate[d] with one another without homogenization” (Galison 1997: 782) or mere translation. The heuristic device which captures these insights is “intercalated periodization” (Galison 1997: 799, figure 9.5).

The theory of TZs focuses on the dynamics and processes of intercalation at the boundaries and intersections between traditions. In fact, Galison argues, close inspection of such sites often reveals an ‘interstitial zone’ or ‘locality of exchange’ where local things happen and language plays a key role. All traditions, he contends, have ‘in-talk’ which allows ‘thick’ exchange between members. They may also develop ‘out-talk’ – characterised by a shift of register – as part of an effort to communicate with others. Actual communication across traditions, however, requires ‘cross-talk’ – also called ‘interlanguage’ or ‘trading language’. If this is present then ‘thin’ exchange can occur. For example, “… physicists of different theoretical persuasion can view a bubble chamber image and still find a thin description upon which they can both agree” (Galison 2010: 44). This cross talk can be understood in terms of lexical complexity and Galison distinguishes between jargons, pidgins and creoles. A pidgin contains a few hundred words. A jargon has fewer and a creole is more complex.

Thus, interlanguages are key to understanding the dynamics of TZs and in relation to them Galison offers some important clarifications. First, they are not (and cannot be) imposed but are produced by interaction. Second, they are not lesser versions of pure languages available elsewhere but registers of interaction which are supple and effective in their zones. Third, “… there is absolutely no teleological guarantee. Not every jargon gets developed into a pidgin; not every scientific pidgin molts into a creole in full bloom” (Galison 2010: 43). In fact, he argues, “It is altogether possible that… fields previously bound, fall apart” (Galison 1997: 805). Finally, interlanguages can have ‘wordless’ elements – shared ‘devices and manipulations’ – like tools, diagrams, pictures and procedures and these can link language and practice in a TZ.

Concepts like cross-talk and jargon are important and we use them below, but some common misunderstandings can be avoided by focusing on the notions of trade and zone. In relation to trade, for example, Galison (1997: 803) says “… it is crucial to note that nothing in the notion of trade presupposes some universal notion of a neutral currency”. In relation to zone he says that it should be understood “… as a social, material, and intellectual mortar” binding traditions together (Galison 1997: 803). TZs are also likely to be unstable and dynamic and can be conflictual. Indeed, “understanding the heat generated at such contact points helps explain who and what the systems actually carve out of the cultural world” (Galison 1995: 37). The world, of course, is made up of many TZs at different stages of development which can be nested or overlapping and every tradition or subculture will be participating in many simultaneously.

In the following we use TZs theory beyond science and normatively. In part this is possible because Galison draws on anthropology and linguistics to understand exchange and these disciplines more often than not focus on non-scientific examples. At the same time, as Biagioli (2009) and others have observed, the domain of science itself is changing in ways that imply the need for such extension and a normative orientation. For example, relative to scientists advancing disciplines in comparative isolation, orientation towards real-world problems, inter-/trans-disciplinarity and multi-stakeholder working have become more important. In fact, a number of scholars have already extended TZs theory as we do. For example, Fuller (2006) and Jenkins (2010) focus on environmental policy and management, and Gorman et al. (2013) focus on the normative introduction of ethicists and social scientists into scientific laboratories. The latter is particularly interesting because it links successful normative use of TZs to the existence of a ‘common goal’.

### Evolution and Authority

Are there different kinds of TZ? How do TZs change or evolve? A number of scholars have engaged with such questions (Collins et al. 2007; Gorman 2002, 2005, 2010, 2011; Gorman and Mehalik 2002; Gorman et al. 2004, Gorman et al. 2009; Jenkins 2010; Kellogg et al. 2006; Mills and Huber 2005; Ribeiro 2007; Balducci and Mäntysalo 2013; Fuller 2006). Their work builds on Galison’s approach and goes beyond by arguing that TZs can pass through different states, classes or stages over time. This can be understood as a journey from no trade to no trade. There is no trade before the TZ forms and no trade will eventually return – either because the TZ evolves into a new and distinct tradition or because exchange ceases to occur at the boundary or intersection. However, on this journey there are many different possibilities.

Drawing on examples such as the development of environmental textiles and earth systems engineering management, Gorman (2005) proposes a three-fold typology of TZ states. In state 1 trading is controlled top down in a hierarchical manner. In state 2 there is compromise and the interests of all actors are balanced through negotiation. In state 3 participants share a mental model which transcends existing boundaries. Collins et al. (2007: 657) build on this but distinguish between TZs depending on “… whether the collaboration is co-operative or coerced and whether the end-state is a heterogeneous or homogenous culture”. Through such frameworks we can begin to conceptualise the evolution of any particular TZ as involving passage through many different states – not only a journey from no trade to no trade but a journey on a meandering path. And it may be that Gorman’s idea of a shared mental model, presumably involving a well-developed inter-language linking linguistic and cognitive dimensions, signposts the demise of a TZ as it evolves into a new and distinct tradition.

Although these frameworks are somewhat different, their authors agree on one important point, which is that expertise plays a key role in the evolution of TZs. Gorman (2002: 934; see also Gorman 2005), for example, contrasts TZs where: (i) there is no shared expertise and “… each discipline tries to dominate the trading zone or threatens to exit”; (ii) there is interactive expertise and “… disciplinary experts create creoles”; (iii) there is contributory expertise “… in which experts… engage each other deeply, learning enough to contribute jointly to development of [something] new”. Collins et al. (2007: 657) agree that “… interactional expertise is a central component of a least one class of trading zone”, but they go further and suggest that such expertise may actually be more important than language in shaping the evolution of TZs. This appears to be a radical departure from Galison’s notion of a TZ, but Collins et al. (2007) do acknowledge the importance of language and Galison (2010: 47) acknowledges the importance of expertise – for example, in the way an ‘engaged interlocutor’ uses both language and expertise.

Operating through or beyond language and expertise are other influences which help to determine how TZs evolve and these may not be under the control of those directly involved in trading – or, perhaps, anyone else. For the purposes of this paper these can be divided into conditions and context. By conditions we mean relatively local or nearby institutional commitments and arrangements. By context we mean wider political, economic and environmental pressures. Galison (2010: 38), for example, discusses ‘external forces’ on TZs like natural disasters and war; the threat or reality of war across the 20^th^ century, for example, encouraged and in some ways required different traditions of physics to work together. In broad terms local conditions can be the vector for contextual factors shaping a TZ. In addition to conditions and context, of course, it is likely that most if not all TZs will be shaped by many essentially unpredictable emergent and contingent influences.

This discussion of different types of TZs and their evolution raises the problem of power and authority. In fact, many scholars who have developed Galison’s work locate language, expertise and other factors in schemes which highlight such things. For example, Gorman (2002, 2005) uses terms like ‘elite’ and ‘egalitarian’ to describe TZs which use expertise in different ways. As we have already noted, Collins et al. (2007) are centrally concerned with coercion and cooperation in TZs, and in relation to the former use the language of ‘colonization’ and ‘hegemony’. In relation to power and authority, and for our purposes, Mills and Huber (2005) make a valuable contribution because they focus on interactions between two academic disciplines – anthropology and education. They identify only limited or one-way interactions, from anthropology to education, and explain this in terms of the relatively low status of education as a discipline, its perceived dependence on particular theories, and the power of hierarchies to structure academia in ways that sustain disciplines often at the expense of interdisciplinary interactions.

## RI in Research Policy

An important milestone in the development of Responsible Innovation (RI) was reached when the EPSRC published its policy on the topic in October 2013 (EPSRC 2013). Prof. Richard Owen (2014a: 113), who played an important role, has described this as “the culmination of a four-year process of collective sense making”. In this section we begin the task of trying to understand how RI is linking applied sciences, social sciences and research funders by describing the key issues and influences which encouraged EPSRC to embrace it, the emerging lexicon of RI and the porous boundary between different variants.

### Issues and Influences

Our research makes clear that one of the key issues which led EPSRC to engage with RI in the early 2010s was the changing nature of the research projects it was funding. As one senior EPSRC employee working in the area of strategy and familiar with RI said:

*[I think in part RI gained traction because of] a dawning realisation that the nature of research that we funded was changing… [it used to be] grounded in theoretical aspects of physics, chemistry, but now we find that our research portfolio embraces potentially disruptive technology such as synthetic biology, nanotechnology, geo-engineering, robotics … [then came the idea that] perhaps what we need to do is also start to think about how we might manage that and how we might encourage our research cohort to manage that … (Interview, December 2012)*

Although RI should not be understood as a purely defensive or reactive move on the part of EPSRC, the reference to ‘disruptive technology’ in this quote includes a concern that accompanying the changing nature of research is an increased potential for public controversy – as illustrated by agricultural biotechnology, which our interviewees often referred to. A second important influence on the uptake of RI was EPSRC’s ability to work across disciplines, including across applied sciences and social sciences. For example, when reflecting on RI, a senior EPSRC employee working in the area of impact made the following point:

*… our funding will stretch into other research council areas as long as it’s not the major share of the work, and that’s helping to bring together a really great multidisciplinary team to tackle a problem. (Interview, December 2012)*

Such issues and influences coalesced and surfaced in different parts of the EPSRC, including the Societal Issues Panel (SIP). The SIP was created in 2006 to “…advise EPSRC Council about how best to take account of public opinion and attitudes in policy development” (EPSRC 2006). It included senior academics from diverse backgrounds and according to one of its members was like a “cross disciplinary research panel” (Interview, January 2012). In 2010, Owen began to present his work on RI to the SIP. This was encouraged by panel members such as Prof. Phil Macnaghten (sociology), Prof. Judith Petts (geography), Prof. Cathy Sykes (physics and public engagement) and Prof. Paul Younger (engineering), and the SIP agreed that it was something that the EPSRC should consider embedding in research policy. Reflecting on this later, Owen emphasised the support of particular panel members and how the SIP helped him to expand his understanding of RI (Interview, November 2012). A senior EPSRC employee working in the area of strategy reflected as follows:

*I suppose at a strategic level, the partnership really happened through the Societal Issues Panel… all of a sudden we brought this group of people together who I’d almost describe as being quite eclectic, who were a cohort of social scientists, ethicists, public engagement and people from our own space. And out of that dynamic we started to see things like public dialogues, responsible innovation, aspects of work on ethics, stuff that was not there hitherto. (Interview, December 2012)*

Connecting this section to the following one on the SPICE project, it is also important to note how the SIP linked RI and geoengineering. A senior EPSRC employee recalled how this happened in an interview:

*It was a coincidence… the geoengineering paper that we provided to our Societal Issues Panel, which drew on and reflected on the [geoengineering sandpit]… was at the very meeting when we were talking about the outcome of a responsible innovation exemplar that we’d done on nanotechnology.*

The final influence on RI at the EPSRC that we want to highlight (drawing on our interviews) is the commitment of particular members of staff, including Chief Executive Professor David Delpy and Senior Business Manager Peter Ferris. The commitment of the first became clear following the UK’s synthetic biology public dialogue which concluded, amongst other things, that research councils had to act in a more responsible way in relation to the research they fund (Bhattachary et al. 2011). Politicians asked Delpy to respond and in doing so he made a public commitment to RI (Delpy 2011: 41–42).

### An Emerging Lexicon

So far we have drawn attention to the changing nature of EPSRC funded research, the organisation’s ability to work across disciplinary boundaries, particular multi-/interdisciplinary institutional arrangements and significant interventions by individuals. However, in focusing on such relatively concrete factors we run the risk of overlooking less tangible ones. The most obvious, given this paper’s engagement with the theory of TZs, is language, and our research confirms that it played an important role and continues to do so.

A useful starting point is to observe that ‘responsible innovation’ is one of many ways of referring to this agenda (or similar). For example, the final report of the DEEPEN project on nanotechnology emphasises ‘responsible development’ (Davies et al. 2009; Ferrari and Nordmann 2009). This is interesting not least because the Principal Investigator, Macnaghten, adopted ‘responsible innovation’ soon after and has since played a significant role in developing the RI agenda within the EPSRC and beyond (see above and later). At the time of writing, ‘responsible development’ has largely been dropped in favour of ‘responsible innovation’, although the former did make some progress in research policy for a time (e.g. TSB/EPSRC 2011). Interestingly, Grunwald (2013) uses ‘responsible research and development’. We discuss another variant, ‘responsible research and innovation’, in the following subsection.

Beyond illustrating that language changes, our research highlights how those involved in RI use it purposefully in particular ways. In relation to ‘responsible’, for example, Owen emphasised:

*… you can’t rely on constructs of responsibility that look back, you have to think about how you can be responsible going forward and what role you will play and they [EPSRC] said, yes, actually we do have a role to play and we understand the public think it’s important we have a role. (Interview, November 2012)*

(Re)defining responsible in this way does work not least because it avoids or bypasses issues of blame and liability – it is prospective not retrospective, shared not apportioned. And, when it is used in this way, the concept has the capacity to link a wide range of actors.

We see something similar in relation to ‘innovation’. An important point here is that advocates of RI use this concept to suggest that implementation of their agenda will open up new – scientific and commercial – opportunities. This helps to counter various criticisms and assumptions that scientists and others might have regarding different forms of oversight, regulation or interference in their work; not least that such things block or stifle innovation (see, for example, Stilgoe’s (2014) response to ter Meulen (2014)). More specifically, in relation to social scientists tasked with implementing RI it counters the idea that they only want to be critics standing outside the process and pointing out why new technologies are unacceptable.

Beyond ‘responsible’ and ‘innovation’ other words are extending the RI agenda. For example, building on the process we discuss in the following section, Stilgoe et al. (2013) proposed ‘anticipation’, ‘reflexivity’, ‘inclusion’ and ‘responsiveness’ as four dimensions of RI – now referred to as ‘AIRR’. In subsequent interactions with EPSRC these dimensions became ‘anticipate’, ‘reflect’, ‘engage’ and ‘act’ or ‘AREA’ in its policy on RI. Thus we see another stage in the process of negotiating and developing the RI lexicon. To date there has been no sustained critical reflection on the lexical shifts involved here although Owen, one of the architects of EPSRC’s policy, has observed that EPSRC ‘felt more comfortable’ with the modified concepts. In addition, he does not think this is problematic because “the substance and meaning of these terms are consistent with the dimensions we developed” (Owen 2014a: 116). Elsewhere, Macnaghten, Owen, Stilgoe et al. (2014) refer to the various RI framings as being ‘interpretively flexible’.

Given the small number of shared words, there is no need to focus on these further but two further points regarding the emerging lexicon need to be made. The first is that the skills or capacities of particular individuals to use language in particular ways have played a role. For example, in the following Owen reflects on his background and how this helped him to work across boundaries and “talk in different languages”:

*[I was trained as an environmental scientist and worked for the Environment Agency on emerging risks]… that’s when I started to get interested in the STS [science and technology studies] literature and particularly in concepts of anticipatory governance and technology assessment and upstream engagement… I’m not a social scientist, so I position myself in a slightly different way, as more of a policy-oriented scientist… I work across different disciplines, I see myself more as a mediator and connector… The point is that you need somebody I think that has a broad overview across different areas and can talk in different languages… so that’s probably where my role has been as a sort of intermediary… (Interview, November 2012)*

Second, the lexicon of RI is not limited to words but includes tools and procedures such as stage-gate review which we discuss below.

### Porous Boundaries

Before moving on to describe the implementation of RI we will briefly explore the boundaries around the debate and interactions across them. This has implications for the unit of analysis and whether or not there is a single RI TZ or many overlapping ones. In the space available we are unable to explore all boundaries, but key questions can be raised by focusing on the interface between RI as discussed above and in the remainder of this paper and Responsible Research and Innovation (RRI), which has gathered momentum at the European Union level particularly in the Framework Programme 7 and Horizon 2020 research funding programmes. The SYNENERGENE project (2013–2017), for example, uses the strapline ‘responsible research and innovation in synthetic biology’.

There are many links, overlaps and similarities between RI and RRI. These are acknowledged by the fact that von Schomberg (2013), one of the key architects of RRI in the European Commission, has a chapter in Owen et al.’s (2013) edited book on RI. However, those associated with RI have also asserted a boundary between these debates and have attempted to exclude aspects of RRI from RI in the UK. Their concerns are set out in Owen et al. (2012: 760):

*Policy statements from the EC suggest that RRI has underlying motivations that are not only instrumental (i.e. in supporting the delivery of policy commitments in the Horizon 2020 Strategy and Innovation Union) but also normative and substantive… If RRI risks becoming a new label for business-as-usual, it also risks being used instrumentally, to smooth the path of innovation in society, and/or to achieve precommitted policies. This, we argue, should be a primary point of discussion and clarification, acknowledging we are at a stage before the term itself becomes locked-in. The purposes and motivations for RRI at a policy level must be clear.*

An example is the way the European Commission has linked RRI normatively to pursuit or furthering of particular ‘European values’ as opposed to making the values which innovation should embody and further part of the debate.

This illustrates a boundary, albeit porous, between RI and RRI. Those promoting RI in the UK acknowledge some elements of RRI whilst at the same time attempting to distance it from other aspects.

## Implementing RI

In the previous section we focused on RI in research policy. In this section we extend the discussion to implementation of RI. Our focus is the SPICE project which was funded by the EPSRC, NERC (Natural Environment Research Council) and the STFC (Science and Technology Facilities Council) to explore one geoengineering response to climate change: releasing particles into the atmosphere to reflect solar radiation with the effect of cooling the Earth. This project initially conceived to involve field testing a balloon delivery system – the ‘test bed’ or ‘balloon experiment’ – and it was this technology which became the focus for implementation of RI by EPSRC in a way that also shaped the development of RI in research policy. Thus, as observed by Stilgoe (2011: 326): “As well as trialling a new technology, [the] SPICE project is a test bed for the idea of ‘responsible innovation’”. We begin by considering the multiple localities where relevant interactions occurred and then move on to discuss how RI was implemented in relation to the balloon experiment – particularly a stage-gate review which concluded ‘pass pending’ when it met.

### Multiple Localities

Towards the end of 2009 the EPSRC, NERC (Natural Environment Research Council) and LWEC (Living With Environmental Change) began to consider organising a sandpit research funding event on climate geoengineering. Those involved recognised the complexity of the issues and the need for a multi- or interdisciplinary group and approach and in some ways were successful in pursuing this vision. For example, the January 2010 Call for Participants states:

*We welcome applications from engineers, climate scientists and climate modellers, as well as from natural, environmental and social scientists. Those engaged in the study of ethics and governance in relation to climate change and the environment are also welcome to apply. Expertise is required from a very broad range of disciplines, so applicants should not feel limited by conventional perceptions: the whole sandpit approach is about bringing together people who would not normally interact.*

Similarly, at the sandpit which was held 15–19 March 2010, the organisers invited Duncan McLaren, Chief Executive of Friends of the Earth Scotland (2003–2011), to help set the scene. He was invited to be ‘provocative’ and took the opportunity to raise the issue of ethics.

In other ways, however, the event failed to implement the multi-/interdisciplinary agenda. For example, only two social scientists were selected – Prof. Nick Pidgeon (social psychology and public engagement including risk and new technologies) and Dr. Maia Galarraga (philosophy and sociology including of technology) – and in an interview one of them said it was unclear what role the funders wanted them to play (November, 2012). Two factors are worth mentioning in relation to this. First, the Economic and Social Research Council had decided not to participate in the sandpit and this may have impacted both on the selection of social scientists and the design of a process which would recognise and facilitate their contribution. Second, sandpit funding events in general are unusual and suit some people and not others in that they involve intense facilitated interactions between participants who must collaborate and compete with each other (often having never met before) for research funds.

These factors help to explain why the climate geoengineering sandpit had limited success in linking the technical and societal issues. This is illustrated in the following quote where a senior academic who participated in the sandpit reflects on the process:

*… there were discussions right at the start about the ethical dimension… But then it kind of got lost… So it’s an interesting reflection that the process didn’t necessarily allow the participants to think about it in those terms… some kind of wider ethical scrutiny… came up right at the final hurdle, when they realised what they’d funded. (Interview, November 2012)*

In this quote the respondent is referring specifically to the SPICE project with Dr. Matt Watson (Bristol University) as overall Principal Investigator and Dr. Hugh Hunt (Cambridge University) as Co-Investigator focusing on the balloon experiment. The sandpit also supported the Integrated Assessment of Geoengineering Proposals (IAGP) project, which we do not discuss here, but one respondent speculated that the balloon experiment attracted support because of its obvious and intriguing engineering challenges.

Although the sandpit process had limited success in linking technical and societal issues, the awareness that the latter were important remained. As a result, the EPSRC added a public engagement process to SPICE after the event (Parkhill and Pidgeon 2011; Pidgeon et al. 2013). This involved a series of micro-dialogues organised by Pidgeon. One interesting aspect of this work is that those involved located it (and themselves) at arms-length from the SPICE project. This was emphasised in interviews and is clear in related publications. For example, Parkhill and Pidgeon (2011: 5) describe their contribution as an ‘extra’ and ‘independent’ work package done at ‘EPSRC’s request’. This positioning is explained by a fear that working closely with SPICE could lead some stakeholders – particularly environmental NGOs – to conclude that the independence of the social scientists had been compromised.

This brief account does not cover all of the settings and processes which linked applied sciences, social sciences and research funders in relation to climate geoengineering at this time. Other examples include meetings of the Royal Society (Royal Society 2009), the 19 October 2009 EPSRC/NERC/LWEC geoengineering scoping workshop (EPSRC/NERC/LWEC 2009), and the development of the Oxford Principles of geoengineering research (Rayner et al. 2009; also see Owen 2014b). However, despite its brevity, our summary does illustrate how complex the interactions were and it raises questions about collaboration (or not) across the research domain.

### Stage-Gate Review

Following the sandpit the EPSRC decided that the SPICE balloon experiment would have to pass a stage-gate review before it was given the final go-ahead. Owen developed the stage-gate criteria and Macnaghten chaired the review panel. In broad terms the design of the process drew on concepts and practices in innovation management and social scientific work around anticipation, reflexivity and deliberation. The stage-gate review took place on 15 June 2011 and involved the SPICE project team being interviewed by Macnaghten (sociology, chair), Brian Wynne (sociology), Duncan Maclaren (civil society), Gordon McFiggans (atmospheric science) and Guglielmo Aglietti (aerospace engineering). EPSRC employees and Owen were observers and Pidgeon was present to provide feedback on the micro-dialogues. The panel assessed SPICE against five criteria (see Macnaghten and Owen 2011; Stilgoe et al. 2013) and the outcome was ‘pass pending’.

We interviewed five of those involved in the stage-gate review (project academics, panel members and observers) and the people we spoke to raised many complex issues. For example, one member of the panel became concerned that the purpose of the stage-gate was to assess a balloon experiment and not a nascent climate geoengineering technology as he had initially thought.

*The first thing that struck me was, this isn’t actually a stage-gate review of a geo-engineering project at all, we’re talking about a balloon 1 kilometre up, see if it’ll float up there and see whether we can anchor it, that’s all… I was very uncomfortable… It’s not going to tell us anything about the extent to which geo-engineering is even feasible let alone acceptable, so what are we doing here [in the stage-gate review]? And then I just thought, it could be lack of vision or it could be very clever deliberate manipulation, who knows… [but] that confusion can’t be allowed to happen… [I emailed EPSRC afterwards to say ‘at the next meeting…] let’s review something meaningful…’ (Interview, November 2012)*

In contrast, another member of the panel focused on how the key conclusion of the review was negotiated.

*We [the panel with EPSRC] had a discussion about whether failure in one of the five [criteria] was enough to fail the whole thing, which I think we agreed, which then unfortunately created a little bit of pressure to put them in the ‘pass pending’ category rather than fail category… I think afterwards, either from their own optimism or from the way it was explained to them… I think they understood ‘pass pending’ as, ‘we’ve passed, we’ve just got to produce these bits of paperwork to show it’, rather than, ‘we will pass if we produce these things… and they are adequate’… As I say, there was some flexibility or ambiguity about the definition of ‘pass pending’, which I think was problematic afterwards. (Interview,December 2012)*

This quote is useful not only because it anticipates later difficulties but also because it draws attention to the work done by ‘pass pending’, particularly in sustaining relationships – between panel and project, across members of panel, between panel and EPSRC, between project and EPSRC – in the context of the stage-gate. There are similarities between this and interpretive flexibility involved when the AIRR framework development by Owen, Macnaghten and Stilgoe was taken up as the AREA framework by EPSRC. We explore this further in the analysis below.

The experiences of the SPICE scientists are also important and underline the challenges. For example, a member of the panel observed that one scientist ‘… was doing his best to be honest, open and flexible, and also to listen to the NGOs who were then on the case’ (Interview, November 2012). Another person present at the stage-gate review observed that one of the scientists:

*… was very very frustrated by the point of the stage-gate to a point almost, ‘why should I be here, this is not my job to answer all these questions’. (Interview, November 2012)*

As an observer Owen described the interaction as follows:

*So, instead of just positioning themselves as scientists producing independent knowledge upon which others would act, you saw these scientists really having to wrestle with these ethical and social issues for the first time, and it was exhausting for them. (Interview, November 2012)*

In his blog, Watson (2011) described it as “a long, exhaustive and exhausting process” but also said it was “fair”, “even handed”, “polite” and “rigorous”.

### ‘Pass(ed,) pending’

Before moving on to our analysis, we will extend the SPICE story beyond where the stage-gate panel concluded ‘pass pending’ in relation to the balloon experiment. A useful starting point is a blog entry written by Watson (2011) the following day, 16 June 2011, which says “The short answer is that the stage-gate was passed, pending some further effort to…”. This is a positive interpretation of the outcome which anticipates the balloon experiment going ahead. Others shared this view (or at least acted on the possibility of it) to such an extent that the University of Bristol, University of Cambridge and NERC issued a press release on 14 September announcing that “The test, the first of its kind in the UK, is expected to take place in the next few months…” (Bristol/Cambridge/NERC 2011). Significantly, the press release argued that those involved were acting responsibly and offered the stage-gate review and public engagement as supporting evidence.

At around the same time others involved in the stage-gate offered more cautious or harder interpretations. A significant example is an article written by Macnaghten and Owen (2011: 293) which was published in *Nature* on 17 November 2011. The article focuses on the “five criteria for responsible innovation” which comprised the stage-gate assessment but does not mention ‘pass pending’, which by this point had become problematic.

*… the panel concluded that although the first two criteria had been met, more was required on the remaining three… When the panel reconvenes, it will independently assess a revised response; until then, the project remains under review.*

The panel, however, did not reconvene. Instead, on 17 October 2012, Matt Watson (the project PI) cancelled the balloon experiment, after meeting with EPSRC and Owen (EPSRC 2012). The update from EPSRC which followed made an explicit reference to RI but in this case (compared with the press release discussed above) to justify cancelling the test bed rather than going ahead with it.

To understand these developments we must locate them in the context of a growing controversy around SPICE which began to gather momentum at the time of the stage-gate review. For example, on 31 August 2011 *The Guardian* newspaper published articles in print and online raising concerns about the balloon experiment. A month later (26 September 2011) an international coalition of NGOs sent a public letter to Chris Huhne MP, then UK Secretary of State for Energy and Climate Change, also raising concerns. Problems also emerged within the project as the team became aware that one of its members had already patented a technology similar to the SPICE balloon (see Ibarrola et al. 2012: 3–5; Owen 2014b). In this paper we are interested in explaining the interactions between applied sciences, social sciences and research funders, so it is enough to merely note these factors and observe that they coalesced into what one of our interviewees representing EPSRC referred to as ‘the SPICE trauma’.

## A Trading Zones Analysis

(Q1) How is RI linking the applied sciences, social sciences and research funders? In the introduction, we argued that analysing RI from a TZs perspective is one way to answer this question. This is possible because building on Galison’s analysis of physics across the 20^th^ century, others have extended the TZs approach both theoretically and empirically, creating a framework which is applicable beyond the internal dynamics of science. In this section we answer Q1 using TZs and focusing on traditions, languages, evolution and authority.

### Traditions and Languages

The heuristic of intercalated periodization is a useful starting point and helps us to make three preliminary observations. First, EPSRC’s engagement with RI encompasses at least three ‘subcultures’ or ‘quasi-autonomous traditions’ of the research domain – applied sciences, social sciences and research funders. Second, in each of these traditions there is evidence of periodisation. The current period, for example, is characterised by increased emphasis on such things as inter-/multi-disciplinarity, the utility of research in relation to ‘grand challenges’, and the economic value of research outputs (cf. Biagioli 2009). Owen (2014a), for example, has described a shift at EPSRC from being a ‘funder’ of research to a ‘sponsor’ or ‘shaper’ of innovation. Third, as illustrated by the stage-gate review of the SPICE project, the traditions are intercalating, or are being intercalated, meaning coordination without homogenization or mere translation (Galison 1997: 782).

Beyond such general points lie insights which can be gained by more subtle application of intercalated periodisation. For example, although the heuristic emphasises that breaks between periods in different traditions do not necessarily coincide, it seems that in our case all traditions are in flux or perhaps beginning new periods of stability simultaneously. Evidence for this is found in the way that the utility of research or inter-/multidisciplinarity, for example, are growing in importance across all traditions at the same time. In fact, we would argue that our example shows not just coordination but also alignment of traditions which is a novel point although not inconsistent with intercalated periodisation. This raises important questions regarding the influences and processes which explain such alignment and the role of RI in these (see below).

These points lay the foundations for a TZs analysis of RI but to go further we must focus on the boundaries or intersections between traditions. When Galison studied these in the context of physics, he found an ‘interstitial zone’ or ‘locality of exchange’ where ‘local things happen’. In our case the interstitial zone is RI; or, perhaps more accurately, RI is creating such a zone where previously there have more often than not only been boundaries/intersections. Interactions between diverse actors in different settings have helped to bring this zone into existence, as illustrated by the EPSRC’s Societal Issues Panel. However, interactions alone do not explain RI. More important is how language has made interactions possible and in so doing has expanded the interstitial zone into a trading zone.

In an interview one senior employee of the EPSRC said that “sometimes we [funders, scientists and social scientists] speak different languages” and to build relationships we must allow “the discourse to happen” (Interview, December 2012). The theory of TZs helps to explain the role of RI in this. Notions like ‘responsibility’ and ‘innovation’ already exist in the ‘in-talk’ of all the traditions involved, but, at the same time, advocates are using both in particular ways to advance the agenda of RI. ‘Responsible’ has been (re)defined as something prospective and collective while ‘innovation’ is used to suggest new opportunities. Both terms, therefore, are available and appealing. In this way ‘responsible innovation’ is providing a ‘thin description’ of challenges and opportunities around science and society and a viable starting point for ‘cross-talk’ which can coordinate different traditions. More normatively it is also providing a shared goal.

A key factor which our case illustrates is the interpretive flexibility of words and concepts. One example is the way that AIRR as proposed by Owen, Macnaghten and Stilgoe, with significant lexical changes, became AREA in EPSRC policy e.g. ‘reflexivity’ to ‘reflection’. Another is the ‘pass pending’ judgement of the stage-gate and its various interpretations. These could be analysed or criticised as examples of ‘fudge’, ‘co-option’ or ‘unseemly compromise’ but to do so would be simplistic. In relation to AIRR-AREA, for example, it is important to remember that TZs involve new languages, not one group simply learning or adopting the language of another. With respect to ‘pass pending’, we would emphasise the diverse interests and expertise of the actors involved in the stage-gate, the related and considerable local coordination challenges and the role that language must play in such a situation. These points are consistent with a TZs analysis but they do not mean we are naive or ignorant of the way language and power can be linked (see below) or the fact that language which is acceptable and even necessary in one setting can become problematic later or elsewhere.

In relation to Q1, therefore, our research shows that RI is linking the applied sciences, social sciences and research funders by expanding boundaries and intersections into a TZ. In this zone language and practice are beginning to bind traditions together encouraging coordination. The TZ is symbolic and conceptual but also exists as particular settings or localities of exchange which are spatial/physical and interactive. However, we should not assume that a new tradition is emerging. At present there is only evidence of local coordination – as illustrated by negotiations around ‘pass pending’ in the SPICE stage-gate. And it is important to emphasise again that there are multiple variants of RI which are overlapping but nevertheless distinct.

### Evolution and Authority

Earlier in this paper we emphasised that TZs can exist in different states and pass through them over time (Gorman 2005). The RI TZ illustrates this. For example, when the EPSRC released its policy on RI in October 2013 it did so in a top down manner (Gorman’s TZ state 1). The policy instructed scientists to apply RI but few had participated in its development or knew anything about it. This contrasts with earlier debates around RI within EPSRC which occurred in a bottom up manner – initiated by Owen and others – with aspects of compromise and negotiation (Gorman’s TZ state 2). To date, however, there is little evidence of transcendence (Gorman’s TZ state 3). This may not be surprising given the limited nature of the RI inter-language and the role such a language must play in a shared mental model.

This line of discussion can be extended by focusing on the nature of collaboration following Collins et al. (2007). For example, the EPSRC’s Societal Issues Panel appears to have been relatively cooperative when compared to the more coercive SPICE stage-gate – the SPICE team had to participate. Somewhere between cooperation and coercion, or forming a corner of a triangle, sits the climate geoengineering sandpit which was competitive in many respects. The simultaneous existence of such diverse collaborations is perhaps just more evidence of the relatively immature nature of the RI TZ and this might change over the longer term – perhaps a narrower range of types of collaboration and/or slower cycling through types over time.

We also emphasised earlier that the theory of TZs has been extended in relation to expertise (Gorman 2002, 2005; Collins et al. 2007) and this provides further insights into the RI TZ. The expertise which features strongly in this case is the ability to work at the interface between research and policy, particularly in relation to science and technology. Owen, Macnaghten and others illustrate this in the way they have advanced RI at policy and project levels. The evidence points to interactional expertise, meaning that those involved know enough to converse with others to promote RI although they may not actually be able to do what others do. This raises the intriguing issue of what role contributory expertise could play in the RI TZ going forward.

Before closing this section we will address the context and conditions within/under which RI is being pursued by focusing on power and authority. Above we speculated on the existence of an overarching process or processes encouraging the alignment of the different traditions in our case and we have highlighted some evidence of RI being implemented at times in a top down perhaps even coercive manner. Thus, we must be prepared to read the RI TZ not in terms of transcendence but in terms of co-option or colonization (following Collins et al. 2007). There are different ways to develop this line of thinking including the way political economy shapes the articulation of science, technology and society (for example, Thorpe and Gregory 2010 on post-Fordism; Moore et al. 2011 on neoliberalism). RI can appear ambiguous in this context. On the one hand, it could open up processes and assumptions but, on the other hand, it could be a product or vehicle of such influences. The most significant parallel we can find in Galison’s work on TZs is the role that war (or threat of it) played in helping to shape and steer all branches of physics across the 20^th^ century. In general, because RI is a relatively young agenda, its political economy has not been explored in detail, but van Oudheusden (2014) is a valuable contribution which focuses on the constitution and contestation of power.

In relation to Q1, therefore, we would emphasise that the RI TZ is passing through different states on a journey from no trade to no trade. This involves different types of collaboration, and expertise is playing an important role. Our research raises questions about power and authority from the dynamics of small groups to the way that political economy is recasting research more broadly. With this in mind we believe that elucidating the links between power and RI should be a research priority. Central to this will be the relationship between the context, conditions and the RI TZ. All analyses, of course, will also have to acknowledge the importance of unpredictable emergent and contingent influences as illustrated by ‘the SPICE trauma’.

## Conclusion

(Q2) How should the policy and practice of RI be taken forward? A useful starting point from a TZs perspective is the observation that interactions and communication between traditions are shaped by contexts and conditions. Although it is unlikely that those attempting to advance RI will be able to change the broader context of science and society, they may nevertheless be able to make use of opportunities which the context provides. Examples include recent controversies around science and technology and widely accepted ‘grand challenges’. Such things can be used to encourage dialogue across boundaries and to open up assumptions around causes and solutions. In relation to local conditions there may be opportunities to secure changes favourable to RI. Organisational support such as endorsement by senior management and financial and physical resources are examples. This is illustrated by the way that Professor David Delpy committed EPSRC to RI in 2011. In a sense this helped to establish the ‘common goal’ for the TZ discussed by Gorman et al. (2013). Recognising contexts and conditions in this way also means, however, that in some situations it will be hard (if not impossible) to advance RI.

Leaving context and conditions to one side our research suggests that the policy and practice of RI should be taken forward by creating additional localities of exchange and opportunities for interaction. Examples from our discussion include the EPSRC’s Societal Issues Panel, the climate geoengineering sandpit and Matt Watson’s blog. Implementing this recommendation over the years ahead might involve organising physical spaces differently, arranging multi-stakeholder meetings or funding collaborative projects. And increasingly there are opportunities for online or virtual interactions of different kinds. This will and should involve experimentation. It is likely, given the stage that RI has reached, that many of these new localities and settings will be at the level of projects (e.g. van der Burg and Swierstra 2013). Drawing on the above, a key consideration should be the nature of power and authority in these new localities because of the way this will shape interactions and communication.

If the normative insights of our analysis were limited to such points, it would add little to what we already know but the TZs perspective takes us further. Drawing on the above, we would also argue that the RI agenda should be advanced by facilitating inter-cultural communication and the building of an RI trading language. Here ‘engaged interlocutor(s)’ might play a key role. Such people may surface unplanned but they can also be trained and appointed. In relation to skills, they should know how to use words, images, objects and diagrams for communication at interfaces between cultures. And it is likely to be helpful if they have topic-specific knowledge – perhaps even contributory expertise. Building an RI trading language – which can be wordless – is likely to be a highly iterative process and should not be understood as simply linguistic/discursive but also cultural and cognitive. Currently the RI lexicon is limited and it will need to become broader and deeper if the RI agenda is to take hold. Some lexical-conceptual shifts which may be useful are available in the literature (Calvert and Frow 2013; Fuller 2013; Fuller and Lipińska 2014), but the theory of TZs emphasises that a trading language must be negotiated and cannot be imposed.

Finally, we would argue that the RI agenda should be taken forward by exploiting opportunities to (reflexively) institutionalise RI, thus changing conditions and perhaps even aspects of context over the long-term. Research-intensive universities may be one of the most important settings for this. They could, for example, create RI units tasked with progressing the agenda in many different ways (see Guston 2007) and in so doing avoid some of the risks associated with implementing RI on a project-by-project basis – such as failure to change institutional culture or ethicists and social scientists losing a sense of their role – ‘going native’ – when they are embedded in laboratories (Gorman et al. 2013; Calvert 2013). There may also be opportunities to open up the infrastructure of research to a wider range of actors and their perspectives, particularly when new buildings and laboratories are being designed. Such institutionalisation would not only change the conditions of RI implementation but may signal a move towards no trade as a new RI tradition emerges.

A recent issue of *Nature* (17 September 2015) focuses on interdisciplinary research and articles explore what governments, funders, journals, universities, academics and others must do to make it happen. Brown et al. (2015) describe 5 key principles such as “forge a shared mission” and “develop ‘T-shaped’ researchers” (meaning broad and deep). Viseu (2015a) makes various suggestions, including that social scientists engaging with scientists should work in teams (not in isolation) and with financial and operational autonomy (see also 2015b). These and other contributions provide insights which could also be used to promote RI in the future. However, in answering Q2 we have shown that a TZs approach provides particular insights which have significant normative implications. This approach does not contradict the work of others but it can provide an additional level of detail and understanding. For example, it helps to elaborate on what might be involved when Brown et al. (2015) stress the need to “forge a shared mission”.

To close we want to emphasise some key insights from the theory of TZs which might help as applied scientists, social scientists, research funders and others wrestle with RI over the years ahead. A TZ is a space where actors from different social worlds communicate and coordinate across boundaries. Central to this is the development of a trading language. But for such a language to emerge those involved need to set aside the idea that interactions involve the corruption or misunderstanding of a pure language and position which exists elsewhere. Instead, the language of the TZ can be understood as a language appropriate to its task, and at least initially a realistic goal for a more extensive RI TZ may simply be better local coordination of the actors with an interest in science, technology and society.
